# High Prevalence of Malnutrition and Sarcopenia with Inadequate Nutritional Support in Intensive Care Unit Patients: A Prospective Observational Study of Clinical Outcomes

**DOI:** 10.3390/nu18060883

**Published:** 2026-03-10

**Authors:** Rym Ben Othman, Asma Cherni, Ismail Dergaa, Halil İbrahim Ceylan, Nagihan Burçak Ceylan, Valentina Stefanica, Ines Sedghiani, Nebiha Borsali, Henda Jamoussi

**Affiliations:** 1 Research Unit on Obesity UR18ES01, Faculty of Medicine, University of Tunis El Manar, 15 Rue Jebel Lakhdar, Beb Saadoun, Tunis 1007, Tunisia; benothmanr@gmail.com (R.B.O.); hendajamoussi@gmail.com (H.J.); 2 Faculty of Medicine Tunis, University of Tunis el Manar, 11 Rue Jebel Lakhdar, Beb Saadoun, Tunis 1007, Tunisia; 3 National Institute of Nutrition and Food Technology of Tunis, 11 Jbal Lakhdar Street, Tunis 1007, Tunisia; asma.cherni.asma94@gmail.com; 4 Higher Institute of Sport and Physical Education of Ksar Saïd, University of Manouba, Manouba 2010, Tunisia; phd.dergaa@gmail.com; 5 Physical Activity Research Unit, Sport and Health (UR18JS01), National Observatory of Sports, Tunis 1003, Tunisia; 6 Department of Physical Education of Sports Teaching, Faculty of Sports Sciences, Atatürk University, Erzurum 25240, Türkiye; 7 Graduate Education Institute, Bayburt University, Bayburt 69000, Türkiye; burcaksehitoglu@gmail.com; 8 Department of Physical Education and Sport, Faculty of Sciences, Physical Education and Informatics, National University of Science and Technology Politehnica Bucharest, 060042 Pitești, Romania; 9 Intensive Care Unit, Hbib Thameur Hospital, Tunis 1008, Tunisia; sedghiani.ines@gmail.com (I.S.); nebiha.borsali@fmt.utm.tn (N.B.)

**Keywords:** critical care, intensive care unit, malnutrition, nutrition, reanimation, sarcopenia

## Abstract

Background: Malnutrition and sarcopenia are highly prevalent in intensive care settings and are associated with adverse clinical outcomes. Aim: The study aimed to evaluate the association between nutritional care, nutritional status, and patient outcomes in intensive care units. Methods: This prospective observational study at a Tunisian tertiary hospital investigated nutritional status and management of 100 intensive care unit patients, each of whom was followed for seven days after ICU admission. Malnutrition Risk was assessed by NUTRIC and MNA scores. The severity of disease was assessed using the APACHE II and SOFA scores. Malnutrition was diagnosed using body mass index and weight loss. Sarcopenia was assessed through grip strength, calf circumference, and psoas muscle area. Nutritional management was evaluated using calculations of caloric and protein intake. Clinical outcomes included the need for intubation, difficulty with oxygen weaning, healthcare-associated infections, and the development of pressure ulcers. Results: The participants had a mean age of 54.85 ± 17.25 years, with a slight male predominance (53 males, 47 females). Pre-existing metabolic conditions affected 80% of patients, including hypertension (40 patients), diabetes (36), and obesity (18). The primary reasons for admission were respiratory disorders (25%), infectious diseases (23%), and metabolic disorders (16%). The mean APACHE II score was 15.91 ± 6.84, and the mean NUTRIC score was 3 ± 1.66; 27% were classified as at high risk of malnutrition. The prevalence of malnutrition reached 50% (28% moderate, 22% severe). Only 31% received adequate caloric intake, while 84% had insufficient protein intake. Malnourished patients required intubation more frequently (50% versus 22.5%; *p* = 0.014), experienced greater difficulty with oxygen weaning (78.4% versus 48.6%; *p* = 0.008), and developed pressure ulcers more often (43.5% versus 6%; *p* < 0.001). Sarcopenic patients showed similar patterns for intubation (51.4% versus 18.9%, *p* = 0.003), oxygen weaning difficulty (77.5% versus 46.9%, *p* = 0.007), and pressure ulcers (39.2% versus 6.7%, *p* < 0.001). Conclusions: Malnutrition and sarcopenia are highly prevalent in intensive care patients and are associated with severe complications, including prolonged mechanical ventilation and pressure ulcer development. Inadequate nutritional support remains common despite known consequences. Early comprehensive nutritional assessment and appropriate management from admission are essential to improve outcomes in critically ill patients.

## 1. Introduction

Critical illness creates profound metabolic stress that threatens nutritional status through multiple mechanisms. Intensive care unit (ICU) patients experience hypermetabolism, accelerated protein catabolism, and impaired nutrient utilization that rapidly deplete body reserves [1]. Healthcare teams appropriately focus primary attention on life-threatening organ failures, yet nutritional status frequently receives inadequate consideration despite its documented impact on recovery [2]. Severe illness induces marked increases in muscle protein breakdown, with losses reaching 150–200% above resting values in polytrauma patients [3]. Fat mass decreases simultaneously, while nutritional interventions often fail to offset these losses [4]. Malnutrition is a state of nutritional imbalance characterized by negative energy and protein balance, which adversely affects physiological function and clinical outcomes [5]. The prevalence of malnutrition among intensive care admissions ranges from 20% to 70% across published studies, with variation attributable to assessment methods, patient populations, and geographic regions [6,7]. This high prevalence carries serious consequences, as malnourished patients demonstrate increased mortality, prolonged mechanical ventilation, higher infection rates, and extended hospital stays compared with adequately nourished individuals [8]. Despite widespread recognition that nutrition is a key determinant of outcomes in critical illness, comprehensive hospital-based data evaluating both assessment adequacy and associations with clinical outcomes remain limited, particularly in low- and middle-income countries [9,10].

Sarcopenia, defined as loss of skeletal muscle mass and function, compounds the nutritional challenges faced by intensive care patients. Critical illness triggers rapid muscle wasting through inflammatory cascades, metabolic derangements, and prolonged immobilization [11]. Patients can lose approximately 2% of skeletal muscle mass daily during the first intensive care week, resulting in a total of 15% muscle loss within seven days [12]. This precipitous decline results from multiple factors, including pro-inflammatory cytokine release, protein catabolism exceeding synthesis, and mechanical unloading from bed rest [13,14]. Sarcopenia prevalence in intensive care populations reaches approximately 41% based on meta-analytic estimates, though rates vary with measurement methods and patient characteristics [15]. Pre-existing sarcopenia serves as a strong independent predictor of adverse outcomes, with one study reporting an odds ratio of 11.87 for early death or chronic critical illness development [16]. Patients with sarcopenia experience increased mortality, prolonged hospitalization, and poor rehabilitation outcomes compared with those maintaining muscle mass [17,18]. Standard sarcopenia assessment methods, including dual-energy X-ray absorptiometry and bioimpedance analysis, are frequently impractical in intensive care settings due to patient immobility and equipment limitations [19]. Alternative approaches using bedside ultrasonography, analysis of existing computed tomography data, and simple anthropometric measures provide practical assessment options [20,21]. Calf circumference is a readily available measure that correlates with muscle mass and predicts clinical outcomes [22]. Psoas muscle area measured on routine abdominal computed tomography is another validated indicator of sarcopenia that imposes no additional patient burden [23]. Handgrip strength assessment using portable dynamometers offers functional evaluation when patients can participate [24].

Optimal nutritional management in intensive care remains incompletely defined despite extensive research. Guidelines from the European Society for Clinical Nutrition and Metabolism (ESPEN) and the American Society for Parenteral and Enteral Nutrition (ASPEN) provide evidence-based recommendations; however, implementation varies widely across institutions [2,25]. Despite the recognized importance of comprehensive nutritional support, many intensive care patients receive inadequate caloric and protein delivery [26]. A gap exists between guideline recommendations and actual practice, with multiple studies documenting substantial underfeeding in intensive care populations [27,28]. This implementation gap reflects multiple barriers, including a lack of protocols, inadequate monitoring, and insufficient prioritization of nutritional care [29]. To address these knowledge gaps and inform local practice, we conducted this prospective observational study to (i) evaluate nutritional status and management of patients hospitalized in intensive care, and (ii) examine the association between nutritional status and clinical outcomes.

## 2. Materials and Methods

### 2.1. Ethical Approval

This study was conducted in accordance with the Declaration of Helsinki. The research protocol was reviewed and approved by the Ethics Committee of the National Institute of Nutrition of Tunis (approval number: 17/2022). Written informed consent was obtained from all participants or their legal guardians prior to enrollment in the study.

### 2.2. Sample Size Calculation

A study by Lobatón [30] examining hospitalized patients reported a malnutrition prevalence of approximately 50%. Based on this prevalence estimate, the sample size was calculated using the formula n = Z^2^p(1 − p)/e^2^, where n represents the required sample size, Z is the Z-score corresponding to 95% confidence level (1.96), p is the estimated event proportion (0.50), and e is the margin of error (0.05). The calculation proceeded as follows: n = (1.96)^2^ × 0.50 × (1 − 0.50)/(0.05)^2^ = 3.8416 × 0.25/0.0025 = 384.16. For a finite population with expected recruitment of 250 patients over one year, finite population correction was applied: n_adjusted = n/[1 + (n − 1)/N] = 384.16/[1 + (384.16 − 1)/250] = 151.3. Considering feasibility constraints and a seven-day follow-up requirement, with an expected 35% exclusion rate (discharge or death before day 7), the adjusted minimum sample size was 96 participants. The target was set at 100 participants to ensure adequate power for planned analyses. Outcome analyses for intubation and oxygen weaning difficulty were conducted on reduced denominators (*n* = 74 and *n* = 72, respectively), comprising patients not yet intubated at Day 0 (*n* = 74) and patients on oxygen therapy at Day 0 (*n* = 72), representing the eligible populations for each respective incident outcome. Post hoc power calculations (two-proportion chi-square, two-tailed α = 0.05) yielded achieved power of 74% and 78%, respectively, based on the observed between-group proportions. Both comparisons were statistically significant, confirming adequate power to detect the reported effect magnitudes, though these estimates fall marginally below the conventional 80% threshold.

### 2.3. Participants

**Figure 1:**
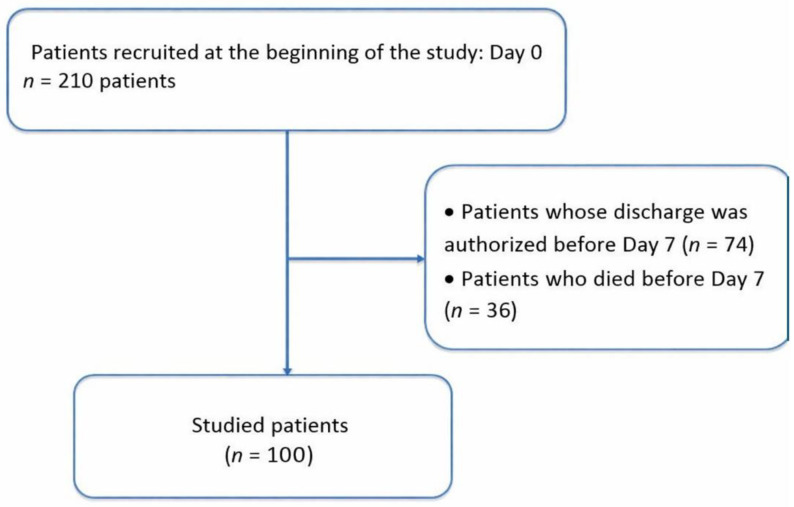
Flowchart of the study.

This prospective, monocentric, longitudinal observational study was conducted in the medical intensive care unit at Habib Thameur Hospital in Tunis, Tunisia, over a-one year period, from 31 March 2022 to 31 March 2023. Adult patients hospitalized in the medical intensive care unit during the study period were eligible for recruitment, regardless of admission diagnosis. Inclusion criteria were: (i) age 18 years or older at intensive care admission, (ii) expected intensive care stay of seven days or longer, and (iii) informed consent obtained from patient or legal guardian. Exclusion criteria were: (i) discharge before the seventh hospitalization day, (ii) death before the seventh hospitalization day, and (iii) refusal of consent by the patient or family. Of 210 patients initially screened, 100 met all eligibility criteria and completed a seven-day follow-up (Figure 1).

### 2.4. Study Procedures

Data were collected on admission day (day 0) and on day 7 of hospitalization using a standardized information sheet. Medical records were reviewed for clinical, anthropometric, biological, radiological, therapeutic, and outcome parameters. All measurements were performed by trained investigators in accordance with standardized protocols.

### 2.5. Anthropometric and Functional Assessment

Body weight was measured using an electronic scale (Beurer, model GS14) for ambulatory patients. For bedridden patients, height was estimated from the heel-to-knee distance measured on a bent leg at 90 degrees, with height calculated using the Chumlea formula adjusted for age and sex [31], and weight was obtained from previously documented medical records; alternatively, family members were asked to provide the most recent known weight, and prior hospital admission records were reviewed for verification. Ideal body weight was determined using the Lorentz formula [32]. Body mass index (BMI) was calculated as weight (kg) divided by height squared (m^2^) [33]. A BMI less than 18.5 kg/m^2^ indicates underweight in adults, while a BMI less than 22 kg/m^2^ indicates underweight in elderly individuals [34]. Percentage weight loss before admission was calculated as [(usual weight − current weight)/usual weight] × 100. Weight loss of 5–10% signaled undernutrition, whereas loss of 10% or more indicated severe malnutrition [35]. Calf circumference (CC) was measured at the widest part of the calf, with values below 31 cm indicating muscle mass loss [24]. Handgrip strength was assessed using an electronic dynamometer (Camry EH108) in conscious, cooperative patients, with thresholds of <16 kg for women and <26 kg for men, in accordance with French National Health Authority guidelines [36]. For patients with abdominal computed tomography imaging, cross-sectional areas of right and left psoas muscles at the third lumbar vertebra level were manually traced, summed, and normalized to height squared to yield total psoas area in mm^2^/m^2^ [23]. Sarcopenia was defined as total psoas area less than 500 mm^2^/m^2^ in men or less than 300 mm^2^/m^2^ in women [23]. Malnutrition was diagnosed if weight loss exceeded 5%, or BMI fell below 18.5 kg/m^2^ (young adults) or below 22 kg/m^2^ (elderly), or sarcopenia was present (defined as handgrip strength below threshold with either calf circumference less than 31 cm or psoas muscle area below normal range).

### 2.6. Nutritional Management Assessment

Nutritional management was evaluated through multiple parameters. The chosen feeding route (oral, enteral, parenteral, or mixed) was documented. The timing of nutrition initiation relative to admission was recorded. For conscious patients receiving oral feeding, dietary intake was surveyed and analyzed using NUTRILOG software (Nutrilog SAS, Paris, France, version 3.2). For enteral and parenteral nutrition, administered quantities were recorded, and caloric, protein, and micronutrient intake were calculated.

The vitamin and micronutrient intake estimates presented are based on manual calculations of actual intake. For patients receiving oral feeding, intake was assessed by recording and quantifying the foods actually consumed, using standard nutritional composition tables. For those receiving enteral nutrition, micronutrient intake was calculated based on the nutritional composition of the prescribed enteral formulas, adjusted to the volume actually administered to the patient.

Resting energy expenditure was estimated using the Harris-Benedict formula, which estimates basal metabolic rate from sex, age, weight, and height [37]. Stress and activity factors were applied to determine total energy expenditure. Caloric and protein targets were adjusted for each patient according to European Society for Clinical Nutrition and Metabolism (ESPEN) and Society of Critical Care Medicine (SCCM) guidelines [38]. Adequate caloric intake was defined as 70–110% of calculated energy requirements. Adequate protein intake was defined as >1.2 g/kg ideal body weight/day [39,40].

### 2.7. Disease Severity and Nutritional Risk Scoring

The Acute Physiology and Chronic Health Evaluation II (APACHE II) score was calculated within the first 24 h of intensive care admission based on physiological parameters, age, and chronic health conditions [41]. The Sequential Organ Failure Assessment (SOFA) score was used to evaluate organ function status [42].

The modified Nutrition Risk in the Critically Ill (NUTRIC m) score was calculated to assess nutritional risk, with scores > 5 indicating high risk [43]. The Nutritional Risk Screening 2002 (NRS-2002) score was applied as recommended by ESPEN [44]. The Mini Nutritional Assessment-Short Form (MNA-SF) was used for elderly participants but has been applied in intensive care settings [2,45].

### 2.8. Laboratory Assessment

Serum biochemical and hematological parameters were collected at baseline. Serum albumin was measured by standard automated methods, and hypoalbuminemia was defined as albumin levels < 35 g/L. Anemia was defined as hemoglobin < 13 g/dL in men and <12 g/dL in women. Hyponatremia was defined as serum sodium < 135 mmol/L, and hypocalcemia as total serum calcium < 2.2 mmol/L.

### 2.9. Clinical Outcomes During the Follow-Up

Clinical outcomes monitored during the seven-day follow-up period included: (i) intubation requirement after admission day for any indication, (ii) difficulty in oxygen weaning defined as inability to discontinue oxygen therapy within seven days, (iii) healthcare-associated infections defined as infections occurring during or resulting from patient management that were neither present nor incubating at management initiation, and (iv) pressure ulcers defined as skin lesions caused by blood supply deficiency due to tissue compression between hard surfaces and bony prominences.

### 2.10. Statistical Analysis

The data were analyzed using SPSS version 26.0 software (IBM Corp, Armonk, NY, USA). Descriptive statistics included absolute and relative frequencies for qualitative variables, and means, medians, standard deviations, and extreme values for quantitative variables. Patient outcomes were compared across several criteria: malnourished versus well-nourished; sufficient versus insufficient caloric intake; sufficient versus insufficient protein intake; sarcopenic versus non-sarcopenic; and those receiving nutritional support versus those receiving exclusive oral feeding. The Kruskal–Wallis H test was used for nonparametric analysis of variance when the distributions were nonnormal. Analysis of variance was used for normally distributed data. Percentage comparisons were conducted using Pearson’s chi-squared test or Fisher’s exact test as appropriate. Statistical significance was set at *p* < 0.05. When multiple independent variables were statistically significant in univariate analysis, multivariable logistic regression models were performed to adjust for potential confounding. For outcome analyses, denominators varied because patients who already had the outcome at admission (e.g., already intubated) were excluded from analyses of incident outcomes (e.g., intubation after admission).

### 2.11. Declaration of Generative Artificial Intelligence Use

Generative artificial intelligence (ChatGPT-4, OpenAI, San Francisco, CA, USA; accessed 28 November 2024) was used solely to verify grammatical accuracy in the final manuscript [46,47]. The authors have carefully reviewed all content, take full responsibility for the scientific integrity and accuracy of the work, and ensure that the manuscript adheres to the highest academic standards. All conceptualization, study design, data collection, statistical analysis, interpretation of results, and intellectual content were performed independently by the authors without the use of artificial intelligence.

## 3. Results

### 3.1. Participant Characteristics

Of 210 patients initially screened, 100 met all eligibility criteria and completed a seven-day follow-up (Figure 1). Participants ranged in age from 18 to 84 years, with a mean age of 54.85 ± 17.25 years. The sample included 53 males and 47 females (sex ratio 1.1). Table 1 presents clinical assessments at admission.

Pre-existing medical conditions were predominantly metabolic (80%), followed by digestive and oncological pathologies. Neurological conditions were also significantly present. Specific metabolic disorders included hypertension (40 patients), diabetes (36), obesity (18), dyslipidemia (32), and hyperthyroidism (6). Renal pathologies affected 16%, primarily chronic kidney disease (12) and nephrotic syndrome (4). Neurological conditions affected 22%, including stroke (13), myasthenia (5), and Parkinson’s disease (4). Surgical history was noted in 10%, involving abdominal surgery (9) and Zenker’s diverticulum (1). Digestive pathologies were present in 24% of cases, including esophagitis (6), celiac disease (6), exudative enteropathy (8), pancreatic insufficiency (2), and hepatic insufficiency (2). Cardiac pathologies were reported in 17%, all with heart failure. Respiratory diseases were present in 13%, with chronic obstructive pulmonary disease (8) and pulmonary hypertension (5).

The primary reasons for intensive care admission were respiratory disorders (25%), infectious diseases (23%), and metabolic disorders (16%). Neurological causes accounted for 13%, including ischemic and hemorrhagic strokes, postictal coma, and status epilepticus. Infectious causes involved septic shock, urinary and pulmonary sepsis, meningitis, tuberculosis, and febrile neutropenia. Metabolic admissions included diabetic ketoacidosis, hyperosmolar coma, myxedema coma, and acute renal failure. Respiratory conditions included chronic obstructive pulmonary disease exacerbations, asthma exacerbations, hypoxemic pneumonia, and pneumothorax. Cardiac-related admissions (12%) included pulmonary embolism, heart failure, and acute pulmonary edema. Gastrointestinal causes (4%) included fulminant hepatitis and gastrointestinal bleeding. Other reasons (5%) included anaphylactic shock, DRESS syndrome, febrile confusion, hunger strike, and polyserositis. Oxygen therapy was administered at admission in 72% of cases.

### 3.2. Anthropometric Profile at Admission

Table 2 presents anthropometric parameters at admission. Weight stability was maintained in 55% of patients. A 5–10% weight loss occurred in 6%, while 3% lost more than 10% in the same period. Over six months, 21% lost 10–15% of body weight, and 15% lost more than 15%.

### 3.3. Laboratory Parameters

Serum albumin levels were measured in 86 individuals, revealing a median albumin of 31 g/L (range 12–47 g/L). Albumin levels below 35 g/L were observed in 78% of patients. Anemia was present in 54%, hyponatremia in 35%, and hypocalcemia in 40%.

### 3.4. Malnutrition and Sarcopenia Diagnosis

Malnutrition was diagnosed in 50% of patients at admission: 28% with moderate malnutrition and 22% with severe malnutrition. The remaining 50% had an adequate nutritional status. Table 3 presents malnutrition diagnosis distribution according to phenotypic criteria.

Overall, 55 patients (55%) were classified as sarcopenic based on composite criteria. Among the 50 malnourished patients, 44 (88%) were sarcopenic. Among the 50 well-nourished patients, 11 (22%) were sarcopenic.

### 3.5. Nutritional Management

Four nutrition modalities were identified. Enteral nutrition was most common (55%), followed by oral feeding (25%), parenteral nutrition (14%), and mixed feeding (6%). Nutrition was initiated upon admission in 18% of patients. Subsequently, 67% began nutrition on day two, while 15% started after day two. Mean estimated energy requirements were 20.49 ± 7.63 kcal/kg (range 15–36.8 kcal/kg). Mean daily energy intake was 1261 kcal/day. Regarding adequacy, 67% of patients received insufficient intake (less than 70% of estimated requirements), 31% had adequate intake (70–110% of requirements), and 2% experienced overfeeding (more than 110%). Mean protein intake was 0.85 ± 0.33 g per kilogram ideal body weight per day, below the recommended 1.2 g per kilogram in most cases. Insufficient protein intake was found in 84% of patients.

Table 4 summarizes vitamin and micronutrient intake during intensive care stay.

### 3.6. Clinical Outcomes

During hospitalization, several notable events occurred. On day 0, 74 patients were not intubated. Among them, 26 (35.1%) required intubation during hospitalization. Regarding oxygen weaning, 72 patients were on oxygen therapy at day 0, while 28 were not. Of the 72 patients on oxygen, 26 (36.1%) were successfully weaned during hospitalization. Healthcare-associated infections were reported in 34 patients (34%). At day 0, 4 patients had pressure ulcers prior to admission. Among the 96 patients without pressure ulcers at day 0, 24 (25%) developed them during hospitalization. Calf circumference was repeated on day 7. Among patients who were not malnourished at admission, 24% had a calf circumference < 31 cm on day 7, whereas it had been normal at admission.

### 3.7. Outcomes According to Nutritional Status

Table 5 summarizes all associations between nutritional status indicators and major complications, including respiratory outcomes, infections, and pressure ulcer development.

Patients presenting with malnutrition or sarcopenia at admission experienced worse evolution, particularly with higher rates of intubation after day 0, greater difficulty in weaning from oxygen, and higher frequencies of healthcare-associated infections and pressure ulcers. Similar trends were observed among patients with elevated NUTRIC m and NRS-2002 scores, indicating that those with higher nutritional risk are more vulnerable to complications.

Conversely, neither the type of nutritional support (exclusive oral feeding vs. enteral/parenteral nutrition) nor the adequacy of caloric intake showed statistically significant differences regarding the outcomes studied. These factors appeared less discriminative in our population.

### 3.8. Multivariable Logistic Regression Analysis

Multivariable logistic regression models were constructed to evaluate whether malnutrition and sarcopenia at admission were independently associated with clinical outcomes (intubation after day 0, difficulty in oxygen weaning, healthcare-associated infection, and pressure ulcer development). Each model was adjusted for age, sex, APACHE II score, and presence of comorbidities. No complication was independently associated with nutritional status or sarcopenia.

For malnutrition, adjusted odds ratios were: intubation after day 0, 9.15 (95% CI 0.18–463, *p* = 0.269); difficulty in oxygen weaning, 8.26 (95% CI 0.15–455, *p* = 0.302); healthcare-associated infection, 1.50 (95% CI 0.08–28.0, *p* = 0.788); pressure ulcer, 7.26 (95% CI 0.27–1916, *p* = 0.486).

For sarcopenia, adjusted odds ratios were: intubation after day 0, 5.33 (95% CI 0.35–82.4, *p* = 0.230); difficulty in oxygen weaning, 7.08 (95% CI 0.34–146, *p* = 0.205); healthcare-associated infection, 1.16 (95% CI 0.19–7.00, *p* = 0.867); pressure ulcer, 4.15 (95% CI 0.25–65.6, *p* = 0.320).

## 4. Discussion

This prospective observational study examined nutritional status, management, and outcomes in 100 intensive care patients over seven days. Three major findings emerged. First, malnutrition and sarcopenia prevalence were remarkably high, affecting 50% and 55% of patients, respectively, with 88% of malnourished patients demonstrating sarcopenia. Second, nutritional management was frequently inadequate: 67% received insufficient calories, and 84% received insufficient protein. Third, malnourished and sarcopenic patients experienced significantly worse clinical outcomes, including higher rates of intubation, delayed oxygen weaning, and pressure ulcer development.

### 4.1. High Prevalence of Malnutrition in Intensive Care Patients

Malnutrition affected 50% of our cohort at admission, consistent with reported prevalence ranges of 20–70% across intensive care populations. Studies examining malnutrition in intensive care show substantial variation depending on assessment methods and patient populations. A study of 287 intensive care patients found that 20.9% had high malnutrition risk scores [9]. Among critically ill elderly COVID-19 patients, the prevalence of malnutrition was 63.4% [48]. In stroke and traumatic brain injury patients, the risk of severe malnutrition exceeded 60% [9]. Several key risk factors contribute to malnutrition. Advanced age is a consistent predictor, with older patients exhibiting significantly higher rates of malnutrition [9]. Mechanical ventilation status, number of comorbidities, and illness severity scores correlate with malnutrition risk [49]. The complex pathophysiology of critical illness creates conditions that favor nutritional deterioration, including hypermetabolism, hypercatabolism, increased protein breakdown, and impaired nutrient utilization [9]. Our findings align with this literature, as 80% of our patients had pre-existing metabolic conditions, and the mean APACHE II score was 15.91, indicating substantial illness severity. The high prevalence of malnutrition underscores the need for systematic nutritional screening upon admission to the intensive care unit. Malnutrition is associated with 40% higher mortality hazard compared with well-nourished patients [8]. Malnourished patients experience longer intensive care stays due to complications, including increased ventilator dependency, higher infection rates, and delayed wound healing [8]. These findings support early identification and intervention as critical components of intensive care management.

### 4.2. Body Mass Index Limitations in Critically Ill Populations

Our study revealed important limitations of relying solely on BMI for malnutrition assessment. While 50% of patients met malnutrition criteria based on BMI, 46% had calf circumference < 31 cm, and 84% had reduced grip strength. These discrepancies highlight that BMI inadequately captures malnutrition in older adults or in critically ill individuals. BMI lacks sensitivity for detecting clinically significant weight losses [50]. A person measuring 1.58 m and weighing 67 kg who loses 10% of body weight would have a BMI decrease from 27 to 24 kg/m^2^, both within the normal range, despite the weight loss being associated with increased mortality risk [50]. Age-related changes, including senile kyphosis, vertebral shortening, and cartilage thinning, reduce stature over time, thereby affecting the accuracy of BMI calculations [50]. Research demonstrates that percentage weight loss is a more effective indicator of malnutrition than BMI or mid-arm muscle circumference [50]. Fluid shifts, edema, and changes in body composition during acute illness can obscure malnutrition when assessed solely with weight-based indices.

### 4.3. Sarcopenia Prevalence and Association with Malnutrition

Sarcopenia affected 55% of our cohort overall, with striking differences based on nutritional status. Among malnourished patients, 88% were sarcopenic, compared with 22% among well-nourished patients. This strong association demonstrates that malnutrition and sarcopenia frequently coexist in critically ill populations. Meta-analysis indicates sarcopenia affects approximately 41% of critically ill patients overall [15]. Sarcopenia serves as a strong independent predictor of adverse outcomes, with one study reporting an odds ratio of 11.87 for early death or chronic critical illness [16]. Critical illness triggers rapid muscle wasting, with patients losing nearly 2% of skeletal muscle per day during the first intensive care week [16]. Rectus femoris thickness decreases approximately 1.75% per day, while cross-sectional area declines about 2.10% per day [12]. This can result in a 15% loss of total muscle mass within one week [12]. Multiple mechanisms drive this rapid muscle loss. Prolonged bed rest and immobilization reduce mechanical loading and muscle activity [11]. Acute illness triggers inflammatory responses that exacerbate muscle wasting through pro-inflammatory cytokines [11]. Critical illness induces metabolic stress, promoting protein catabolism while reducing protein synthesis [14]. Nutritional deficiencies further accelerate the decline in muscle mass and function [14].

### 4.4. Inadequate Nutritional Management

Despite the recognized importance of nutrition in critical illness, our study revealed substantial gaps between nutritional requirements and actual delivery. Only 33% of patients received adequate caloric intake (70–110% of requirements), while 67% received insufficient intake. Protein delivery was even more inadequate, with 84% of participants receiving less than the recommended 1.2 g/kg/day. These findings align with published literature documenting widespread underfeeding in intensive care populations [26,27,28]. Several factors contribute to inadequate nutritional delivery. Feeding interruptions during procedures, gastrointestinal intolerance, and clinical instability frequently prevent the target from being achieved [51]. Lack of standardized protocols and insufficient monitoring contribute to underfeeding [14]. In our cohort, only 18% of patients initiated nutrition upon admission, with 67% starting on day two and 15% after day two. Guidelines recommend early enteral feeding within 24–48 h of intensive care admission [52]. Delayed initiation of nutrition therapy misses opportunities to prevent nutritional deterioration during the acute phase. Protein requirements in critically ill patients range from 1.2 to 2.5 g per kilogram daily, depending on illness severity [40]. Our observed mean protein intake of 0.85 g/kg fell substantially below the recommended intake. Protein-energy malnutrition accelerates during critical illness due to marked increases in protein breakdown. Research in traumatic brain injury patients documented total body protein losses of 1.62 kg (15.5%) over 21 days, with 67% attributable to skeletal muscle [53]. Severely polytraumatized patients lose approximately 2 g of muscle protein per kilogram of body weight per day during the first week [3]. Inadequate protein provision fails to offset these losses, contributing to progressive nutritional deterioration. Micronutrient deficiencies were universal in our cohort; vitamins A, C, E, B2, B5, B6, B9, and D, as well as minerals calcium, phosphorus, potassium, magnesium, and selenium, were insufficient in 100% of patients. Micronutrients play essential roles in immune function, wound healing, and metabolic processes [54]. Their widespread deficiency likely contributes to adverse outcomes observed in malnourished patients. These findings emphasize the urgent need for improved nutritional management protocols. Strategies should include early initiation of nutrition, standardized feeding protocols, regular monitoring of intake adequacy, and attention to micronutrient provision alongside macronutrients.

### 4.5. Malnourished and Sarcopenic Patients Experience Worse Clinical Outcomes

Our study demonstrated strong associations between nutritional status and clinical outcomes. Malnourished patients required intubation more frequently (50% versus 22.5%, *p* = 0.014), experienced greater difficulty with oxygen weaning (78.4% versus 48.6%, *p* = 0.008), and developed pressure ulcers more often (43.5% versus 6%, *p* < 0.001) compared with well-nourished patients. Sarcopenic patients showed similar patterns. These findings align with extensive literature demonstrating that malnutrition is associated with increased mortality, prolonged mechanical ventilation, higher infection rates, and extended hospital stays [8,55]. The mechanisms linking malnutrition to poor outcomes are multifactorial. Muscle weakness from protein-energy malnutrition impairs respiratory muscle function, prolonging ventilator dependence and delaying extubation [55]. Immune dysfunction associated with malnutrition increases susceptibility to infection [8]. Impaired wound healing and tissue repair contribute to the development of pressure ulcers [56]. The dramatically higher-pressure ulcer incidence in malnourished (43.5%) versus well-nourished (6%) patients illustrates malnutrition’s impact on tissue integrity. Multivariate analysis in our study showed no independent association between nutritional status and complications, even after adjustment for confounding variables. This finding contrasts with univariate results and some published literature. Several explanations merit consideration. First, our relatively small sample size (n = 100) may have limited statistical power to detect independent associations after adjustment. Second, complex interactions between nutritional status, illness severity, and outcomes may not be fully captured by available analytical approaches. Third, the seven-day follow-up period may have been insufficient to observe longer-term consequences of malnutrition. Despite the absence of independent associations in multivariate analysis, the strong univariate associations and consistency with the published literature support the clinical importance of malnutrition. The NUTRIC score, which integrates nutritional risk with illness severity, showed associations with outcomes. Patients with NUTRIC scores of 5 or greater had significantly higher rates of intubation (56.5% versus 25.5%, *p* = 0.010), infections (51.9% versus 27.4%, *p* = 0.022), and pressure ulcers (48% versus 15.5%, *p* = 0.001) than lower-risk patients. These findings support the use of validated nutritional risk scores to identify patients requiring intensive nutritional support and monitoring.

### 4.6. Strengths and Limitations

Several strengths characterize this study. First, the prospective design allowed standardized data collection and follow-up. Second, a comprehensive nutritional assessment incorporated multiple modalities, including nutritional scores, physical examinations, laboratory tests, and sarcopenia assessment. Third, this is the first study in Tunisia to assess sarcopenia among patients in intensive care. Limitations warrant consideration. The sample size (*n* = 100) and follow-up duration (seven days) were limited. A single-center design may limit generalizability to other settings. The inability to use indirect calorimetry, considered the gold standard for determining energy requirements, is a significant limitation. Energy requirements were estimated using the Harris-Benedict formula rather than measured directly. Despite these limitations, the study provides valuable data on nutritional status, management practices, and clinical outcomes in a Tunisian intensive care population.

## 5. Conclusions

Malnourished and sarcopenic patients experienced significantly higher rates of intubation, oxygen weaning difficulty, and pressure ulcer development. These findings emphasize the critical importance of early, comprehensive nutritional assessment and appropriate management from the time of intensive care admission. Systematic screening with validated tools can identify high-risk patients who require intensive nutritional support. Standardized feeding protocols, early initiation of nutrition, and regular monitoring of intake are essential to reduce the gap between nutritional requirements and actual delivery. Attention to protein and micronutrient provision alongside caloric targets may improve outcomes. Future research should examine whether intensive nutritional interventions can modify the association between malnutrition and adverse outcomes in critically ill patients, with longer follow-up periods to capture mortality and long-term recovery outcomes.

## Figures and Tables

**Table 1:** Clinical Assessment at ICU Admission (Day 0) (n = 100).

Parameters	Value
Age (years)	54.85 ± 17.25
Male, *n* (%)	53 (53%)
Female, *n* (%)	47 (47%)
Intubated at admission *n* (%)	26 (26%)
Non-invasive ventilation *n* (%)	27 (27%)
APACHE II score	15.91 ± 6.84
SOFA score	4.00 ± 3.16
NUTRIC m score	3.00 ± 1.66
NRS-2002 score	3.00 ± 2.00
MNA-SF score	8.33 ± 4.21

The data are presented as mean ± standard deviation (SD) for continuous variables and number (percentage) for categorical variables. APACHE II: Acute Physiology and Chronic Health Evaluation II; SOFA: Sequential Organ Failure Assessment; NUTRIC m: modified Nutrition Risk in the Critically Ill; NRS-2002: Nutritional Risk Screening 2002; MNA-SF: Mini Nutritional Assessment–Short Form.

**Table 2:** Anthropometric Profile of Patients at ICU Admission (*n* = 100).

Parameters	Mean ± SD
Usual weight (kg)	72.00 ± 23.00
Current weight (kg)	67.00 ± 21.50
Weight loss (%)	7.35 ± 9.00
Height (cm)	165.00 ± 12.25
BMI (kg/m^2^)	24.43 ± 9.43
Calf circumference (cm)	31.72 ± 5.37
Grip strength (kg)	11.32 ± 8.20

The data are presented as mean ± standard deviation (SD). BMI: body mass index.

**Table 3:** Prevalence of Malnutrition Based on Phenotypic Diagnostic Criteria Used by HAS (*n* = 100).

Phenotypic Criterion	Patients Meeting Criterion, *n* (%)
BMI < 18.5 (young adult) or BMI < 22 (elderly)	50 (50%)
Weight loss before admission	45 (45%)
Reduced grip strength *	25 (84%)
Low psoas muscle area index (CT-based) **	27 (75%)
Calf circumference < 31 cm	46 (46%)

The data are presented as a number (percentage). * Grip strength was assessed in 30 patients. ** Psoas muscle area index was available in 36 patients with abdominal CT imaging. BMI: Body Mass Index; CT: Computed Tomography; HAS: Haute Autorité de Santé.

**Table 4:** Vitamin and Micronutrient Intake during ICU Stay (*n* = 100).

Parameters	Mean ± SD	Patients with Insufficient Intake (%)
Vitamin A (µg)	869.58 ± 554.24	100
Vitamin C (mg)	126.21 ± 68.42	100
Vitamin E (mg)	13.70 ± 8.50	100
Vitamin B1 (mg)	1.25 ± 1.16	56
Vitamin B2 (mg)	1.74 ± 1.28	100
Vitamin B3 (mg)	7.66 ± 7.20	60
Vitamin B5 (mg)	3.25 ± 2.41	100
Vitamin B6 (mg)	1.81 ± 1.16	100
Vitamin B12 (µg)	2.93 ± 2.39	99
Vitamin B9 (µg)	299.26 ± 170.87	100
Vitamin D (µg)	11.69 ± 5.82	100
Calcium (mg)	912.22 ± 481.57	100
Phosphorus (mg)	816.17 ± 395.63	100
Magnesium (mg)	301.55 ± 104.08	100
Iron (mg)	15.63 ± 10.91	84
Selenium (µg)	62.35 ± 36.31	100
Sodium (mg)	1721.74 ± 589.92	64
Potassium (mg)	2347.50 ± 813.31	75

The data are presented as mean ± standard deviation (SD) and percentage (%). Insufficient intake was defined according to European Society for Clinical Nutrition and Metabolism (ESPEN) and Society of Critical Care Medicine (SCCM) guidelines.

**Table 5:** Clinical Outcomes According to Nutritional Status and Nutritional Risk Indicators (*n* = 100).

Parameters				*p*
**Malnutrition at Day 0**	No (*n* = 50)	Yes (*n* = 50)		
Intubation after Day 0	22.5% (9/40)	50.0% (17/34)		0.014
Difficulty in weaning from oxygen	48.6% (17/35)	78.4% (29/37)		0.008
Healthcare-associated infection	26.0% (13/50)	42.0% (21/50)		0.091
Pressure ulcer	6.0% (3/50)	43.5% (20/46)		<0.001
**Sarcopenia**	Yes (n = 55)	No (n = 45)		
Intubation after Day 0	51.4% (19/37)	18.9% (7/37)		0.003
Difficulty in weaning from oxygen	77.5% (31/40)	46.9% (15/32)		0.007
Healthcare-associated infection	41.8% (23/55)	24.4% (11/45)		0.068
Pressure ulcer	39.2% (20/51)	6.7% (3/45)		<0.001
**Nutritional Support**	Enteral/Parenteral (*n* = 74)	Oral feeding (*n* = 26)		
Intubation after Day 0	39.6% (21/53)	23.8% (5/21)		0.199
Difficulty in weaning from oxygen	61.0% (36/59)	76.9% (10/13)		0.352
Healthcare-associated infection	33.8% (25/74)	34.6% (9/26)		0.939
Pressure ulcer	28.2% (20/71)	12.0% (3/25)		0.103
**NUTRIC m Score**	<5 (n = 73)	≥5 (n = 27)		
Intubation after Day 0	25.5% (13/51)	56.5% (13/23)		0.010
Difficulty in weaning from oxygen	59.6% (31/52)	75.0% (15/20)		0.223
Healthcare-associated infection	27.4% (20/73)	51.9% (14/27)		0.022
Pressure ulcer	15.5% (11/71)	48.0% (12/25)		0.001
**NRS-2002 Score**	0–3 (*n* = 43)	4 (*n* = 20)	5–7 (*n* = 37)	***p***
Intubation after Day 0	27.1% (13/48)	55.6% (5/9)	35.1% (8/17)	0.131
Difficulty in weaning from oxygen	52.3% (23/44)	100.0% (12/12)	68.7% (11/16)	0.009
Healthcare-associated infection	28.6% (18/63)	50.0% (7/14)	39.1% (9/23)	0.260
Pressure ulcer	9.7% (6/62)	50.0% (7/14)	50.0% (10/20)	<0.001
**Caloric Intake**	Insufficient (*n* = 67)	Sufficient (*n* = 31)	Overnutrition (*n* = 2)	***p***
Intubation after Day 0	30.8% (16/52)	47.6% (10/21)	0.0% (0/1)	0.299
Difficulty in weaning from oxygen	70.5% (31/44)	57.7% (15/26)	100.0% (2/2)	0.091
Healthcare-associated infection	38.8% (26/67)	25.8% (8/31)	0.0% (0/2)	0.266
Pressure ulcer	24.6% (16/65)	24.1% (7/29)	0.0% (0/2)	0.724

The data are presented as a percentage (number of events/numbers at risk). Denominators vary across outcomes because patients who had the event at admission were excluded from analyses of incident events during ICU stay. NUTRIC m: Modified Nutrition Risk in the Critically Ill; NRS-2002: Nutritional Risk Screening 2002.

## Data Availability

The data that support the findings of this study are available at https://doi.org/10.6084/m9.figshare.29913824.

## Abbreviations

The following abbreviations are used in this manuscript:

BMI	Body Mass Index
CC	Calf Circumference
CT	Computed Tomography
ICU	Intensive Care Unit
PMA	Psoas Muscle Area
